# Analysis and visualization of disease courses in a semantically-enabled cancer registry

**DOI:** 10.1186/s13326-017-0154-9

**Published:** 2017-09-29

**Authors:** Angel Esteban-Gil, Jesualdo Tomás Fernández-Breis, Martin Boeker

**Affiliations:** 1 Fundación para la Formación e Investigación Sanitarias de la Región de Murcia, Biomedical Informatics & Bioinformatics Platform, IMIB-Arrixaca, C/ Luis Fontes Pagán, n° 9, Murcia, 30003 Spain; 2 Dpto. Informática y Sistemas, Facultad de Informática, Universidad de Murcia, IMIB-Arrixaca, Facultad de Informática, Campus de Espinardo, Murcia, 30100 Spain; 3 Institute for Medical Biometry and Statistics, Medical Center – University of Freiburg, Faculty of Medicine, University of Freiburg, Stefan-Meier-Str. 26, Freiburg, 79104 Germany

**Keywords:** Biomedical informatics, Semantic web, Cancer registry, Ontology

## Abstract

**Background:**

Regional and epidemiological cancer registries are important for cancer research and the quality management of cancer treatment. Many technological solutions are available to collect and analyse data for cancer registries nowadays. However, the lack of a well-defined common semantic model is a problem when user-defined analyses and data linking to external resources are required. The objectives of this study are: (1) design of a semantic model for local cancer registries; (2) development of a semantically-enabled cancer registry based on this model; and (3) semantic exploitation of the cancer registry for analysing and visualising disease courses.

**Results:**

Our proposal is based on our previous results and experience working with semantic technologies. Data stored in a cancer registry database were transformed into RDF employing a process driven by OWL ontologies. The semantic representation of the data was then processed to extract semantic patient profiles, which were exploited by means of SPARQL queries to identify groups of similar patients and to analyse the disease timelines of patients.

Based on the requirements analysis, we have produced a draft of an ontology that models the semantics of a local cancer registry in a pragmatic extensible way. We have implemented a Semantic Web platform that allows transforming and storing data from cancer registries in RDF. This platform also permits users to formulate incremental user-defined queries through a graphical user interface. The query results can be displayed in several customisable ways. The complex disease timelines of individual patients can be clearly represented. Different events, e.g. different therapies and disease courses, are presented according to their temporal and causal relations.

**Conclusion:**

The presented platform is an example of the parallel development of ontologies and applications that take advantage of semantic web technologies in the medical field. The semantic structure of the representation renders it easy to analyse key figures of the patients and their evolution at different granularity levels.

## Introduction

Cancer registries are an important part of the health information systems in local and regional health organizations. Regional and epidemiological cancer registries are the foundation for cancer research and the quality management of cancer treatment. In most developed countries, the operation and the sampling of data in cancer registries are statutory. Cancer registries are complex structures for the documentation and analysis of data from patients diagnosed with cancer [1, 2]. Different types of cancer registries collect patient data from institutions (institutional), regions (regional) or complete larger areas (epidemiological). Whereas epidemiological registries provide mainly population-based information on morbidity and mortality, institutional and regional registries can provide fine-grained information on treatment and conditional survival.

The information of regional cancer registries serves different requirements such as the quality control of patient care, the comparison of patient-related outcome parameters and research support. Institutional and regional registries are also the main data source for epidemiological cancer registries. Regional cancer registries collect information about diagnosis, therapies and course of the disease [3], the most important being the histopathology of the primary tumor, including tumor staging and grading. The long-term follow-up of the patients’ vital status is one of the resource-intensive tasks of tumor registries providing the basis for survival analysis.

Different software cancer registries solutions are currently available, such as METRIQ^1^, OncoLog Registry^2^ or CNEXT^3^. The standardisation of the cancer registry software is difficult because of a large set of rapidly changing legal and scientific requirements. Most of these software solutions suffer from two main limitations. The interoperability with other health applications such as Electronic Medical Records (EMRs) is limited, which is a typical problem of clinical information systems [4]. The heterogeneity of the underlying data models is a consequence of the difference between data models in current cancer registry software [5, 6]. This imposes severe limitations on research and on the progress of cancer studies when clinical research activities need to integrate data from different cancer registries of several regions.

There have been proposals to overcome the afore mentioned problems. In [7] the authors use the Unified Modelling Language for modeling cancer registry processes in a hospital. In [8] the authors propose a set of indicators to evaluate specific quality measures in cancer care, and [9] attempts to optimise cancer registries by means of knowledge-based systems for monitoring patient records. Unfortunately, these approaches do not guarantee the generation of standard models and do not provide satisfactory solutions to scenarios which require customisable, comparative analyses and data linking to external resources [5].

On the technical side, the Semantic Web stack can be employed to provide information with given well-defined meaning, better enabling computers and people to work in cooperation [10]. Ontologies [11] constitute the standard knowledge representation mechanism for the Semantic Web, in which languages such as the Web Ontology Language (OWL) enable a formal representation of the domain of interest. Important international initiatives [12, 13] strive to ensure that the Semantic Web becomes a fundamental system to achieve consistent and meaningful representation, access, interpretation and exchange of clinical data. These semantic web technologies have already been used to represent cancer diseases, e.g. in [14], an ontology models clinic-genomic cancer trials. Ontologies were also proposed to represent certain types of cancer disease [15, 16].

The main objective of this study is the development of a Semantic Web platform that facilitates the analysis and visualisation of data from cancer registries including (1) the representation of the disease course of a patient, (2) the representation of the aggregated disease courses of a group of patients, and (3) the definition of customisable dashboards for patient selection and visualisation of the data. The use of simulated data demonstrates the viability of incorporating a local cancer registry into this model. A comparative performance analysis of relational databases and semantic repositories demonstrates excellent performance measures for the semantic repository.

## Background

### Standards and classification systems in cancer registries

Most information contained in cancer registries is derived from primary care interactions. For the purpose of structured secondary documentation, tumor documentaries carefully reprocess primary documentation. In many countries, a standardised common dataset has been developed to better support exhaustive data exchange with the epidemiological cancer registries, proposing the classification of diagnostic and treatment information with clinical coding systems.

The most important clinical classification system applied in cancer registries is the International Classification of Diseases version 10 (ICD-10) [17]. This classification system is divided in chapters, with blocks of diseases. For example, chapter II includes the classification for neoplasms between the blocks C00 and D48. These blocks are subdivided in hierarchies that further specify the diagnosis. The ICD-O is a domain-specific extension of ICD for cancer diseases. ICD-O is a dual classification allowing the coding of topography (tumor site) and tumor morphology. SNOMED CT [18] has adopted ICD-O codes for the classification of tumor morphology.

Several staging systems for cancer have evolved over time and continue to evolve with scientific progress. The most important classification system is the Classification of Malignant Tumours (TNM) [19], which is related to the description of the anatomical extent of the disease. This system is under constant development by the Union for International Cancer Control and the American Joint Committee on Cancer. The TNM staging is based on the size or the extent of the primary tumor, the metastases in regional lymph nodes, and the presence of metastasis or secondary tumors formed by the spread of cancer cells to other parts of the body.

Clinical procedures are also encoded with coding systems such as the ICD10-PCS (Procedure Coding System) [20] denoting aspects such as the clinical classification of the procedure, the surgical section or the body system.

### Visualisation of clinical records

From the emergence of the electronic medical record (EMR), the amount of data has increased exponentially [21, 22]. The main objective of the EMR is representing the clinical characteristics of a patient from several perspectives. For a variety of reasons [23] this objective has not yet been achieved.

Visualisation methods are one way of facilitating the representation and flexible exploitation of EMR data. According to [24] there are two types of visualisation of EMR data:

- Multimedia visualisation includes video, audio, graphical plots, rich text, hyperlinks and other multimedia contents [25, 26].
- Temporal visualisation depicts clinical timelines of the health state of the patient [27, 28]. Some of these representations are able to generate a prospective of the future clinical characteristics of the patient using data mining techniques over all the EMR [29, 30].

The *TimeLine* project [24] combines the two approaches with four key aspects of the user interface: demographics and encounter information, medical problem list, graphical timelines and the data viewer that allows the navigation over all data of the patient as bone scan, laboratory data, etc. The main advantage of this project is that the clinician can visualise all patient data without switching between various information systems.

### Semantic exploitation of data

#### Semantic representation

The methods for the transformation and semantic representation of information follow similar approaches. They can be classified in (1) those which generate a representation of the datasets in semantic formats being the result of the application of mappings between the entry data source and the ontology that provides the meaning for the content; and (2) those which permit ontology-based data access using data in traditional formats but querying with semantic web query languages. Next, we describe the most popular approaches and tools from both categories:

- **D2RQ (Accessing Relational Databases as Virtual RDF Graphs)** allows to query data stored in relational databases using SPARQL on virtual RDF graphs [31]. This tool is totally automatic.
- **Triplify** allows to publish [32] the content of relational databases as Linked Data [33] based on a partially automatic transformation process.
- **Linked Data Views (Virtuoso).** OpenLink Virtuoso [34] is a database management system that handles several persistence models (relational, XML, object-relational, virtual and RDF). Persistence models stored in Virtuoso can be queried with SPARQL based on the automatic representation as Linked Data Views [35].
- **XS2OWL (Representation of XML Schemas in OWL syntax).** XML schemas can be transformed into OWL [36]. XML databases can be automatically transformed and queried with SPARQL.
- **RDB2OWL (A Database-to-Ontology Mapping Language and Tool).** Approach to transform the data stored in relational databases into RDF or OWL [37]. The user manually defines mappings between the entries and the outputs. The transforming of large ontologies can be tedious.
- **Karma.** It links a source model to ontologies to generate a semantic representation of the data source [38]. This process is partially automatic.
- **Populous.** Assistant for building ontologies [39], the process being guided by patterns. Populous is able to import CSV data.
- **SWIT (Semantic Web Integration Tool).** Semantic transformation engine capable of generating RDF and OWL repositories from both relational and XML databases [40]. Besides transforming the data, SWIT prevents the generation of logically inconsistent data with the support of DL reasoners. The transformation method has three main steps: (1) definition of the mapping rules between the fields of the database and the ontology; (2) generation of the OWL data; and (3) importing the OWL data into the semantic data store.

Most approaches are based on the mappings between the relational and semantic primitives of the corresponding models languages. Performing only a syntactical transformation, the meaning of the content is not really exploited. In this work we use the SWIT transformation approach, which preserves the meaning of the content based on the specification of mappings between the entities of the source relational schema and the entities of the target domain ontology.

#### Semantic querying

The amount of RDF data, and the development of applications that use semantic web technologies for storing, publishing and querying data has increased constantly in the last decade [41]. Semantic endpoints in which the users can exploit the data without any knowledge of SPARQL have been developed. For example, Natural Language Processing has been used to develop a question answering system [42]. In other works, the authors use parametrised queries to answer questions based on a template [43]. In faceted search over RDF repositories, the user can refine the filters over the results of each SPARQL query [41].

In the biomedical field, the use of semantic querying is limited to the generation of semantic searchers or dashboards. BioDash is an example of semantic dashboard that exploits heterogeneous data sources for drug discovery [44]. Chem2Bio2RDF provides dashboards automatically collecting associations within the systems’ chemical biology space [45]. In this work, our goal is to go beyond the state of the art by allowing users to dynamically define their semantic dashboards.

## Methods

### Ontology construction

Best practices in ontology engineering recommend to reuse existing content and to create modular ontologies [46]. These recommendations are implemented reusing concepts from different ontologies so that the resulting ontology infrastructure is likely to be a networked ontology. The OBO Foundry has also developed a series of principles for ontology construction which propose principles for modularity, orthogonality and reusability [47].

The method for constructing the domain ontology used in this work consisted in identifying the main entities that should be represented, searching BioPortal for existing ontologies containing classes representing these entities, selecting the most appropriate ones (by our subjective criteria), and extending them when necessary. The final ontology has been implemented using Protégé^4^ in OWL-DL, which is the OWL subset based on Description Logics.

### Data generation and representation

In this work, we have generated a simulated cancer registry dataset using the statistical distribution of a real registry dataset, following the method proposed in [48]. Data provided by the National Cancer Registry of Ireland^5^ were used to obtain a patient distribution by age. The cancer registry was accessed on 10-05-2016 and we included 533409 cases diagnosed from 1994 to 2013. The patients were generated in groups classified by gender and 5-years age ranges (0-4, 5-9, 10-14, etc.). The last group of patients contains people older than 85 years old.

**Fig. 1:**
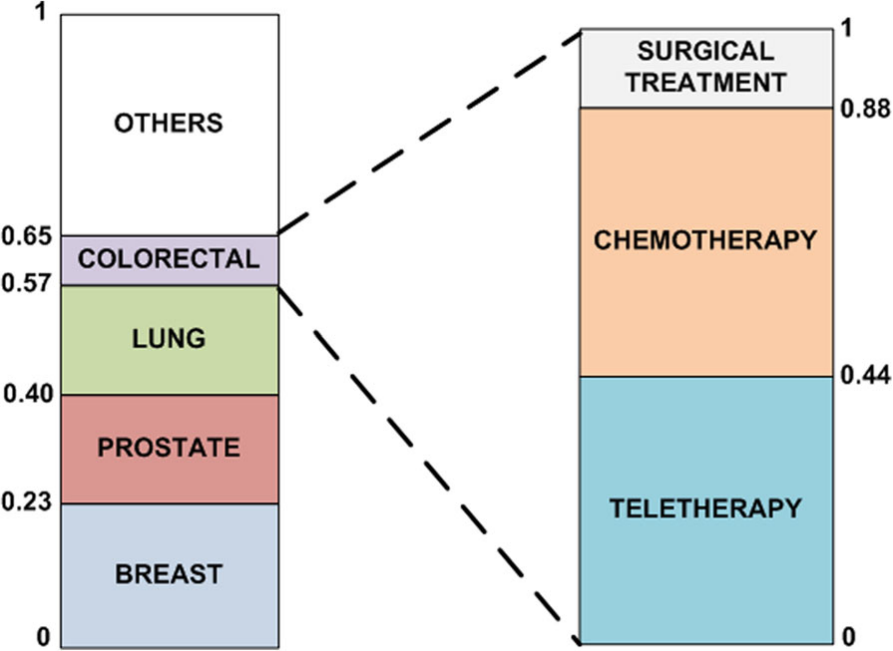
Schema of probability distribution of diagnoses and therapies

For each group of patients we have calculated the probability distribution of diagnosing a concrete type of cancer, and the probability distribution of receiving a particular therapy (surgery, chemotherapy, radiotherapy, hormonal therapy,...) for a concrete diagnosis. These probabilities were used to assign weights to every type of cancer with its therapies for each group of patients. For example, for patients between 60 and 64 years old, the probabilities for different types of cancer are breast cancer (0.23), lung cancer (0.17), prostate cancer (0.17), and colorectal cancer (0.08). For patients within this age range and diagnosed with colorectal cancer the probabilities of the therapies would then be: teletherapy (0.44), chemotherapy (0.44) and surgical treatment (0.12). Figure 1 shows the stack of distributions. When the random number is between 0.57 and 0.64 we assign colorectal cancer as the patient’s diagnosis. Then, we generate a new random number to assign the first therapy and so on.

Furthermore, survival and mortality data were used for extracting the evolution of the disease. Finally, we ensured that the amount of patients with more than one cancer diagnosis meets the distribution of the real dataset.

Our simulated dataset consists in randomised cases. For each case, we establish the gender and age of the patient. Then, we apply a partially random distribution algorithm for getting the patient characteristics. This algorithm uses the weights assigned to each type of cancer, therapy or course to generate distributions similar to the original database. This algorithm is able to generate patients with one or more diagnoses with various therapies and courses following the probability distribution previously calculated.

Such data have been represented in RDF by applying SWIT, whose transformation method has three main steps: (1) definition of the mapping rules between the database schema and the ontology; (2) generation of the RDF data; and (3) importing the RDF data into the semantic data store. We use a semantic repository to store the data, which integrates two types of data sources: (1) an OWL files server with the formal representation of the domain, and (2) an RDF repository which stores the data. Virtuoso^6^ is used as data store [49].

### Exploitation model

Our approach includes a set of methods for exploiting the information model in the semantic repository.

#### Ontology-driven search (ODS)

SPARQL is the language used for querying the data store. We use our ontology-guided input text subsystem [50] to make it easier for clinicians to exploit the data warehouse. The main objective is to allow users to design and execute SPARQL queries without knowing SPARQL. This tool is an editor for SPARQL queries supported by an OWL ontology. The OWL ontology provides the classes and properties that can be used for creating the SPARQL query that will be executed on the RDF repository. The construction of the queries begins with the selection of a main class of the ontology. For example, if we wish to find patients, then the ODS begins with the selection of the ontology class Patient. The user can define filters over this class by using the data properties or object properties of the ontology. The use of *owl:ObjectProperty* permits to include other concepts in the query. For example, if we wish to find patients whose diagnosis is lung cancer, the user can select the *owl:ObjectProperty* *hasDiagnosis*, which is associated with the class *Patient*, which permits to use the *owl:ObjectProperty* Pathological structure of the class *Diagnosis* to select the class representing lung cancer. The ODS is able to generate SPARQL queries in which the subject is an ontology class, the predicate is a property and the object can be either a value or other concept. By selecting an *owl:ObjectProperty*, the user can add other properties of this concept to the query. This service follows the approach of template-based searches [43].

With this tool, the data store can be searched using the properties defined in the ontology. Moreover, it allows the generation of aggregated queries for the elaboration of representative charts of the data store. The generated queries can be stored for parameterisation and reuse. Aggregate functions such as count, average, min or max can be used.

The results of these queries can be linked with other resources. The filters used can also be stored for later reuse. The semantic search engine not only allows for data retrieval but also for creating new classes in the semantic model, which can be assimilated to OWL defined classes. For example, the query for patients with colon cancer could be defining the class “Patient with colon cancer”. The members of this class are obtained by executing the corresponding query.

#### Semantic profiles

Conceptually speaking, the semantic profile is defined as the set of relations and properties of an individual. Semantic profiles permit to identify groups of patients that share the same properties and are therefore useful for comparing and studying such groups. Ontologies are of special interest for creating profiles because they allow to select and aggregate individuals from a conceptual perspective. Our approach can also generate the semantic profile of a group of patients by applying one or more criteria.

Hence, we define a semantic profile as the subset of semantic information of an individual that is interesting for a particular analysis. The profile of the individual *i* is calculated as shown in Eq. 1.

1 $$ SP(i) = S(d) \cup S(SP(o))  $$

where *S*(*d*) represents a subset of the selected *owl:datatypeProperty* and *S*(*SP*(*o*)) represents a function that retrieves the individuals linked through *owl:objectProperty* axioms to *i*. The semantic profile is built by the application of the ODS by using the entities defined in a domain ontology. The ODS permits to select the properties of interest and to define the filtering and aggregation conditions. The user can define the SPARQL queries that will return the subset of properties and relationships that provides the best description of the individual for the specific case. This information is obtained for each individual, and the results can be viewed as a cache of the most important semantic information describing the individuals.

Semantic profiles can be seen as a purpose-specific application of the semantic search engine. Two types of semantic profiles are of special relevance in the context of this work, namely, the timeline representation of a patient and the aggregated disease timeline representation of a patient group with some common properties. Both are described in the next sections.

#### Disease timeline of a cancer patient

**Fig. 2:**
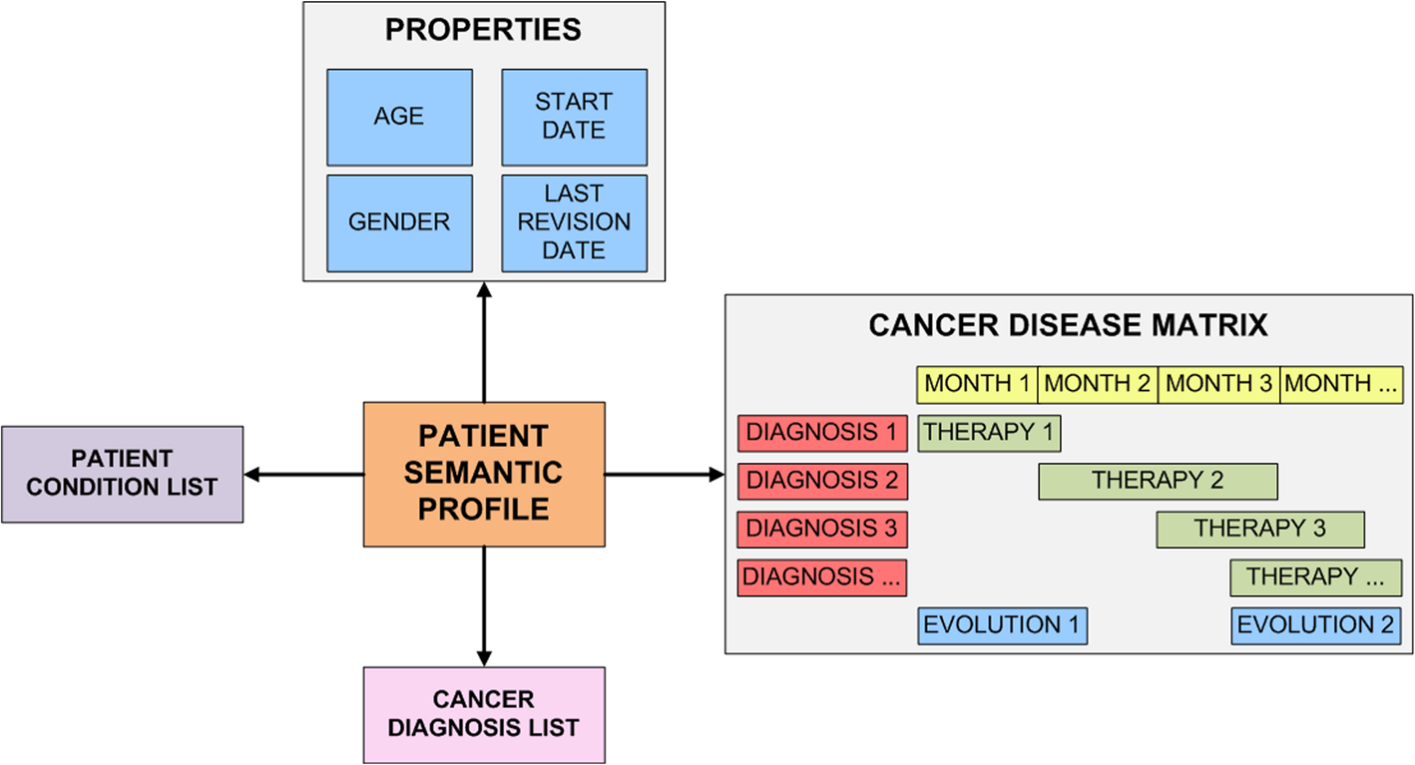
Schema of semantic profile of a cancer patient

The disease timeline of a patient contains information about various health-related events (e.g. diagnosis, patient conditions, therapies and the disease courses). Retrieving these events for a patient requires data normalisation for the representation of therapies by month. Figure 2 shows that every diagnosis has an associated timeline which includes therapies and the disease course, both ordered by month. For example, we can show the timeline for a breast cancer patient that includes the applied therapies (surgical treatment, chemotherapy, etc.) for every period. Furthermore, we can show the course of the disease and its relation with changes in therapies. It also includes the date of the diagnosis and the date of the last encounter. Finally, the profile contains all the patient’s diagnoses and a list of her conditions.

#### Aggregated disease timeline of a group of patients

**Fig. 3:**
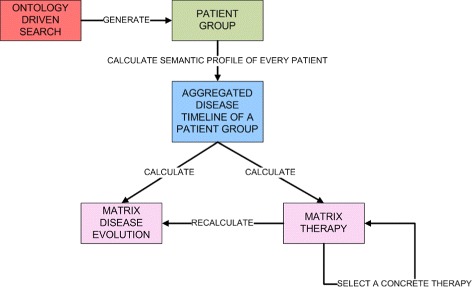
Overview of the generation of aggregated disease timeline of a patient group

The aggregated timeline of a patient group (see Fig. 3) includes all the events of the selected patients who have the same selection criteria for a given period and for a concrete diagnosis. The groups of patients are defined using the ODS, which permits to define groups of patients with the same diagnosis, staging, grading and age range. This permits to obtain the semantic profile of each member of the group. Then, the semantic profiles of the members of the group are globally analysed, so obtaining a matrix that contains the disease courses of the included patients for every month of the disease. Using this method, the user is able to generate, for example, a group of patients with lung cancer with ages between 60 and 70 years old. In this case, our service could represent which therapies are applied in chronological order and which are the most likely courses. At the same time, these graphical representations can be used as new filters to recalculate the corresponding variables. For example, if the user selects to apply chemotherapy as first therapy, the representation changes to reflect the new scenario.

#### Enrichment analysis

Enrichment analysis is a type of statistical analysis that is frequently used in biomedical domains [51]. Our enrichment analysis method is based on the hypergeometric distribution method established for the GO:TermFinder to determine the significance of a Gene Ontology annotation to a list of genes [52], and the hypergeometric distribution was developed using Apache Commons Math^7^.

This type of analysis is useful to compare several subsets of patients with the same diagnosis. We perform a statistical analysis of the ICD-10 codes to support the users in the definition of diagnosis-based groups. We calculate the *P*-value for each group as shown in Eq. 2.

2 $$ P = 1 - \sum_{i=0}^{k-1}\frac{\binom{M}{i}\binom{N - M}{n - i}}{\binom{N}{i}}  $$

where *N* is the total number of ICD10 codes used in the cancer registry, *M* is the number of diagnoses annotated with each ICD10 code, *n* is the number of ICD10 codes of interest for a concrete patient group and *k* is the number of ICD10 codes used for annotating each diagnosis.

#### Semantic dashboard

A semantic dashboard is a graphical representation of the results of one or more queries. Semantic dashboards are represented as 〈〈*L*, *V*〉, *isDashboard*, *U*〉 where 〈*L*, *V*〉 are the results of the SPARQL as key-value pairs 〈*L*, *V*〉, and *U* is who defined the dashboard. Each user can define and customise her dashboards.

The semantic dashboard is implemented using the ODS and permits to create aggregated data. The results can be represented graphically and in tabular format. Based on the persistence model of SPARQL queries, the representations can be used for accessing the data instances contained in each representation. Consequently, aggregation control boxes can be regarded as search filters of the semantic search engine.

**Fig. 4:**
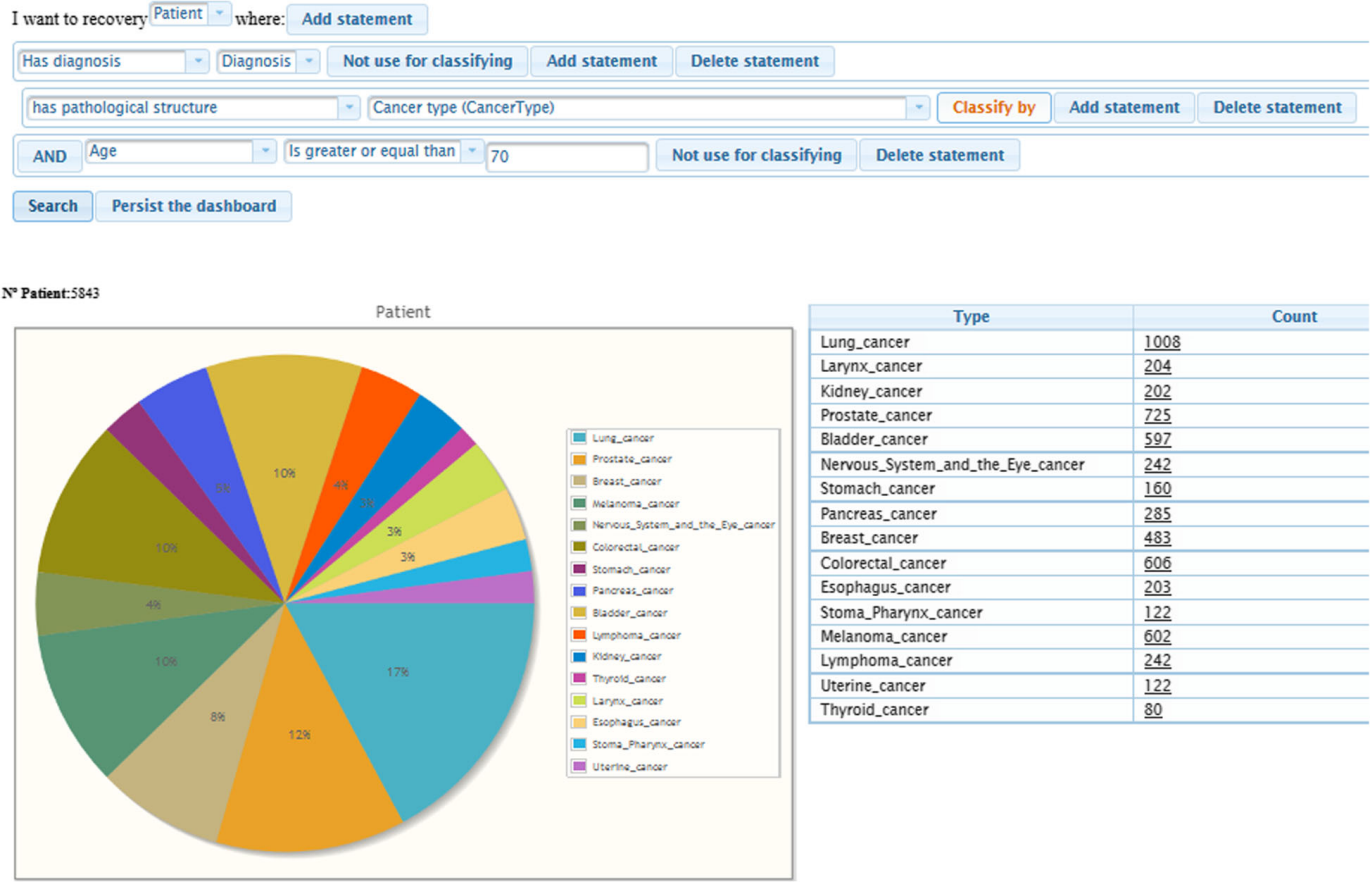
Example of semantic dashboard

Figure 4 shows the query generated with the ODS for searching patients over 70 years old and classified by cancer type. In the left side we show the graphical representation and in the right side the data in tabular format.

The semantic dashboards can also include multiple aggregated queries and display comparative graphics. Finally, dashboards can also be persisted, parameterised by users and reused.

#### Recommendation

We have developed an algorithm based on Bayesian networks to suggest the most appropriate treatment for a patient. This algorithm is based on the generation of probabilistic models using semantic nodes profiles. Bayes networks cannot have cycles [53], but our semantic dataset might contain cycles. The semantic profiles might have cycles due to, e.g., the repetitive application of a given treatment to the patient. To solve this problem a tree network is generated for each profile.

In case of being interested in knowing which treatment is likely to be the most appropriate for a patient given a number of features, the model would first retrieve all the patients with such features, and then use their semantic profile to generate the map of Bayesian networks with the possible treatments by period (month, term, etc.). Once a treatment is selected, the network is re-calculated to improve the next recommendation. Given this dynamic aspect of the network, the method requires that the user indicates which characteristics might generate a cycle in the network to prevent the algorithm from falling in an infinite loop.

## Results

The approach described in the previous section has been applied in a scenario that simulates an institutional cancer registry. An ontology modeling the semantics of an institutional cancer registry has been developed. This ontology has driven the transformation of the simulated dataset into RDF and its storage in the semantic data store. We have implemented a Semantic Web platform that permits users to exploit the cancer registry dataset by formulating incremental, customisable queries using a graphical user interface based on the ODS and by generating dashboards on demand. The complex timelines of the disease of individual and aggregated patients can also be explored and analysed. Next, more details about these results are provided.

### The ontology

We have built a preliminary cancer registry ontology^8^ based on the existing ontologies and fulfilling the requirements of a local cancer registry. This first draft ontology represents some aspects of cancer diseases and their treatment pragmatically. The ontology reuses the Semanticscience Integrated Ontology (SIO) [54] and the Ontology for Biomedical Investigations (OBI) [55]. The ontology incorporates concepts from clinical standards used in cancer such as ICD10, ICD-O-3, TNM staging, Karnofsky index [56] and ASA index [57]. The ontology has been defined in OWL-DL. The metrics of the ontology are as follows (numbers in brackets represent the number of entities added by our work). The ontology contains a total of 20,551 classes (335), 28 properties (18) and 342 object properties (29), with 152,529 logical axioms (2581). The ontology defines the following classes:

- *Patient* represents a person with any type of cancer disease. Properties: gender, birth date, diagnosis, therapies and disease courses. This class is equivalent to the class *Patient* in SIO.
- *Patient condition* represents the health condition of a patient at a given time. Properties: reference date, age, weight, height, Karnofsky index, ASA index and the menopause status.
- *Diagnosis* represents the patient diagnosis at a given time. Properties: ICD10 code, grading, staging, therapies, date, pathological structure, anatomical structure and tumor type. This class is equivalent to the class *Diagnosis* in SIO.
- *Therapy* represents the patient therapies of a diagnosis at a given time. Different kinds of therapy such as *Chemotherapy*, *Surgical Treatment*, *Nuclear Medicine* and others have been modeled in the ontology as subclasses of Therapy. Properties: medication, start date and end date.
- *Disease course* represents the development in time (process) of a tumor disease of a certain type (diagnosis) over a time interval at a given time point. Different kinds of course such as *Complete remission* (tumor is not detectable any longer), *Progression* (tumor mass increases to a certain amount), *Recurrence* (after complete or partial remission, tumor mass increases again), and others have been modeled in the ontology as subclasses of *Disease course*. Properties of disease course are diagnosis, patient conditions, stage, order and date. The properties date and order are the key to sort the courses of the patient for a concrete diagnosis. This class is equivalent to the class *Disease course* in OBI.
- The ontology also includes some classes to represent the TNM classification system of malignant tumors. They include anatomical entities for cancer grading and staging, e.g. *Primary tumor*, *Regional Lymph Nodes* and *Distant Metastasis* hierarchies.
- Health Classification System is the superclass of all classes representing coding artifacts of health related classification systems. To build the taxonomies of classifications for a cancer registry, we tried to reuse other ontologies. For the ICD10 code we use the ontology built in [58].

We have evaluated the quality of our ontology using the Ontology Quality Evaluation Framework (OQuaRE) [59]. OQuaRE is a framework for evaluating the quality of ontologies based on standards of software quality. OQuaRE automatically calculates quality scores in the range [1,5] for a series of characteristics and subcharacteristics. A score 1 indicates that it does not fulfill the minimal requirements, 3 indicates that the ontology meets the requirements, and 5 indicates that the ontology exceeds the requirements. Table 1 shows the results for our ontology. The scores for Functional Adequacy, Maintainability, Operability, Structural and Transferability are over 4. The lowest results are achieved for Compatibility and Reliability, although they are over 3. The results show that our ontology has a high level of cohesion, consistency, formalisation, modularity and reusability, which are the most relevant aspects for the present work.

**Table 1:** OQuaRE Metrics for the Cancer Registry ontology

Subcharacteristic	Value
Compatibility (3.25)	
Replaceability	3.5
Functional Adequacy (4.69)	
Clustering and Similarity	4.5
Consistent Search and query	4.8
Controlled Vocabulary	5.0
Guidance and Decision Trees	5.0
Indexing and Linking	4.67
Infering	5.0
Knowledge Acquisition	4.67
Knowledge Reuse	4.875
Reference Ontology	4.5
Results Representation	3.5
Schema and Value Reconciliation	4.75
Text Analysis	5.0
Maintainability (4.34)	
Analysability	4.33
Changeability	3.86
Modification Stability	4.0
Modularity	5.0
Reusability	4.5
Testeability	4.33
Operability (4.83)	
Learneability	4.83
Reliability (3.0)	
Availability	4.0
Recoverability	2.0
Structural (4.67)	
Cohesion	4.0
Consistency	5.0
Formal Relation Support	4.0
Formalisation	5.0
Redundancy	5.0
Tagledness	5.0
Transferability (4.25)	
Adaptability	4.25

### The semantic cancer registry system

**Fig. 5:**
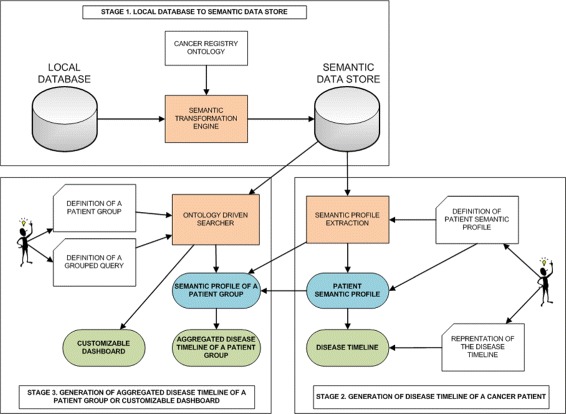
Overview of the system

We have implemented a prototype system^9^ based on the methods described in previous sections. Figure 5 shows the three main parts of this system. All the components of our system have been developed from scratch except SWIT, which is a previous result of our research group. The upper part of the figure shows the data transformation module, which uses SWIT for transforming the original data in semantic information stored into the semantic data store.

The cancer registry ontology is the core of the system, allowing for the computational management of the information related to the cancer patients. All the services offered by the prototype are implemented on top of this core. The data transformation requires to map the source data schema to the cancer registry ontology.

The lower part of the figure shows the other two modules of the system. The right one shows the module for the analysis of individual patients, that is, extraction of semantic profile and timeline analysis. The left one shows the module for the analysis of groups of patients, which also includes the graphical access to the disease courses of those groups. The ODS permits to create groups of patients that share some semantic properties. This permits to generate charts and tables with accumulated data of the semantic repository. In this case, the system provides an option for adding the grouping class or property, so that it can be considered as a customisable dashboard designer. The dashboard permits users to select and aggregate the information on every class of the semantic model. This module is the base for the construction of other services such as the graphical representation of the aggregated timelines of a group of patients or the customisable dashboards.

The dashboard visualises the concepts of the model in charted and grouped forms, and multiple, on-demand, incremental dashboards can be built. For instance, a user can generate a pie chart selecting patients by their first therapy. The user can save any dashboard for querying the results without needing to generate it again.

### Application to the simulated dataset

We have performed an initial evaluation of the system. We have generated a simulated database with 207.190 patients^10^. By the application of SWIT, the generated dataset meets the constraints defined in our ontology, whose entities are used for creating the RDF dataset. The time for the transformation of the dataset from the relational database to the semantic datastore has been thirty-two minutes (Main features of the server: Intel *Ⓡ* Core^TM^ i7-3770T Processor (8M Cache, up to 3.70 GHz), 8GB RAM, SATA2). We have carried out some tests based on the execution of different types of queries to compare the performance of the relational and semantic stores.

Table 2 ^11^ shows that the time performance of the semantic datastore is slower than the relational one for basic queries that do not require joins. However, the semantic datastore performs better than the relational model, even with indexes, on this dataset for more complex queries. The semantic datastore is also faster when filtering by a single property of the class or the table column.

**Table 2:** Results of the migration of the relational database to the semantic data store

Query	SQL	SQL	SPARQL	SPARQL
	count	time	count	time
	result		result	
Recovery all Patients	207.190	0,060s	207.190	0,189s
Recovery all Therapies	400.290	0,132s	400.290	0,317s
Recovery all Diagnosis	240.088	0,070s	240.088	0,220s
Recovery all Courses	108.297	0,030s	108.297	0,155s
Recovery patients with diagnosis, therapies and courses	207.190	1,048s	207.190	0,204s
Recovery all female Patients	105.714	0,231s	105.714	0,189s
Recovery all female Patients with more of 60 years old	62.603	0,245s	62.603	0,192s

#### Semantic dashboard

**Fig. 6:**
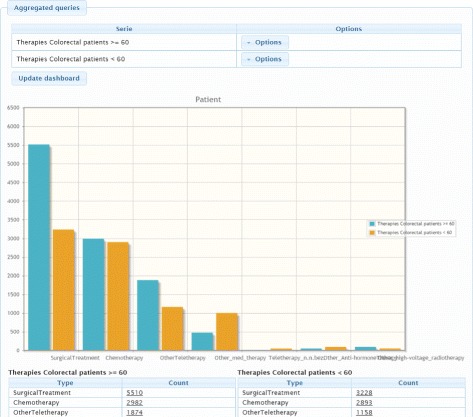
Dashboard view

This tool permits users to formulate incremental, user-defined queries with a graphical user interface based on the ODS. Figure 6 shows a comparative graphic over the therapies applied to patients diagnosed with colorectal cancer in different age ranges. Table 3 shows the generated query for this case. The query results can be displayed in several customisable ways, allowing for the generation of on-demand dashboards.

**Table 3:** SPARQL query generated by ODS for a dashboard

PREFIX ods: 〈http://www.imib.es/ontologies/disease-times 〉
SELECT count(DISTINCT ?s), ?t WHERE
{{?s rdf:type ?t FILTER (?t IN (ods:DrugTherapy, ods:Anti-hormoneTherapy,
ods:Anti-hormonal_anti-androgens, ods:Anti-hormonal_anti-estrogens,
ods:Anti-hormone_therapy_aromatase, ods:Other_Anti-hormoneTherapy,
ods:Chemotherapy, ods:Immunotherapy, ods:OtherdrugTherapy,
ods:Bisphosphonates, ods:Other_med_therapy,
ods:NuclearMedicineTherapy, ods:OpenRadionuclides,
ods:Other_nuclear_medicine_therapy, ods:RadioiodineTherapy,
ods:OtherTherapy, ods:Hyperthermia, ods:Locoregional_hyperthermia,
ods:Part-body_hyperthermia, ods:LightTherapy, ods:OtherLightTherapy,
ods:Selective_ultraviolet_phototherapy, ods:Wait_and_see,
ods:Radiotherapy, ods:Brachytherapy, ods:Interstitial_brachytherapy,
ods:Other_brachytherapy, ods:OtherHigh-voltageRadiotherapy,
ods:High-voltage_radiotherapy_n.n.bez.,
ods:Other_high-voltage_radiotherapy, ods:Whole-body_irradiation,
ods:Teletherapy, ods:OtherTeletherapy, ods:Teletherapy_n.n.bez.,
ods:Teletherapy_with_linear_accelerator, ods:StemCellTransplantation,
ods:AllogeneicSCT, ods:AutologousSCT, ods:SurgicalTreatment,
ods:Therapy))}.
{{?s ods:hasDiagnosis ?a0.
{?a0 rdf:type ?ta0 FILTER (?ta0 IN (ods:Diagnosis))} }.
{?a0 ods:hasPathologicalStructure ?a01.
{?a01 rdf:type ?ta01 FILTER (?ta01 IN (ods:Colorectal_cancer))} }.
{?s ods:hasPatient ?a1. {?a1 rdf:type ?ta1 FILTER (?ta1 IN (ods:Patient))} }.
{?a1 ods:age ?a12. FILTER (?a12 〉= 60)} }} group by ?t}

#### Graphical representation of the disease timeline of a patient

**Fig. 7:**
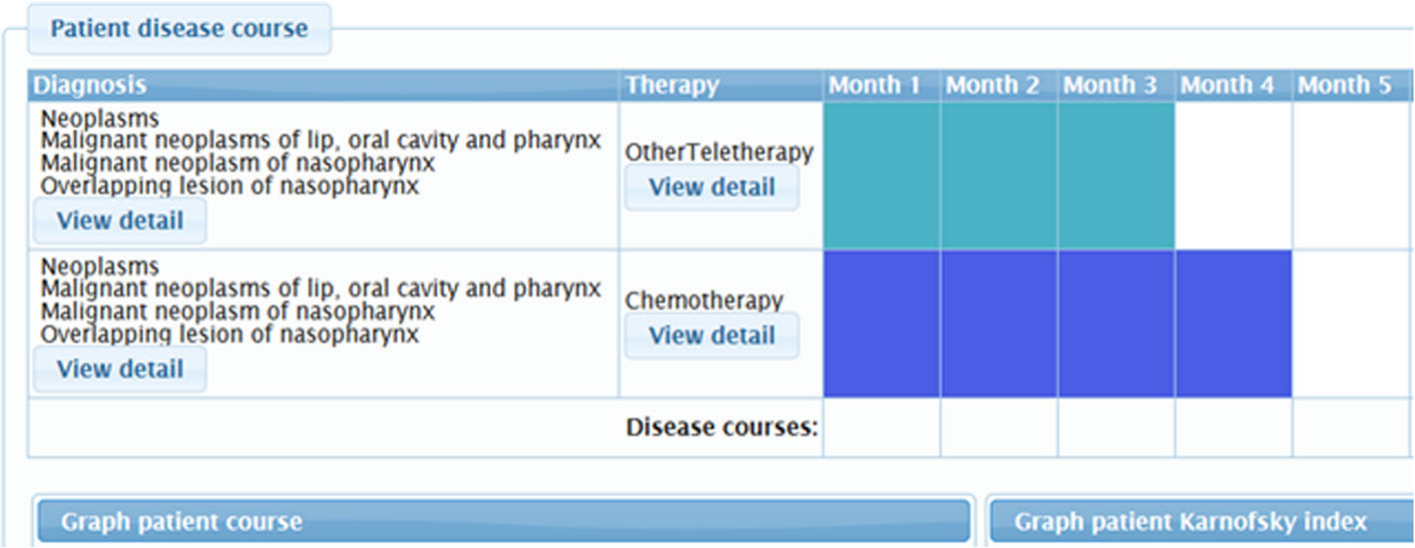
Excerpt of the timeline representation

This service permits users to observe the main properties of the timeline of a patient with a cancer disease. Figure 7 shows an excerpt of the therapy and course timeline of a patient with pharynx cancer. In this view, users can see the details of the diagnosis and of every therapy applied in each period. Besides, users are provided with two evolution charts, which are based on the patient course and on the Karnofsky index.

#### Graphical representation of the aggregated disease timeline of a patient group

**Fig. 8:**
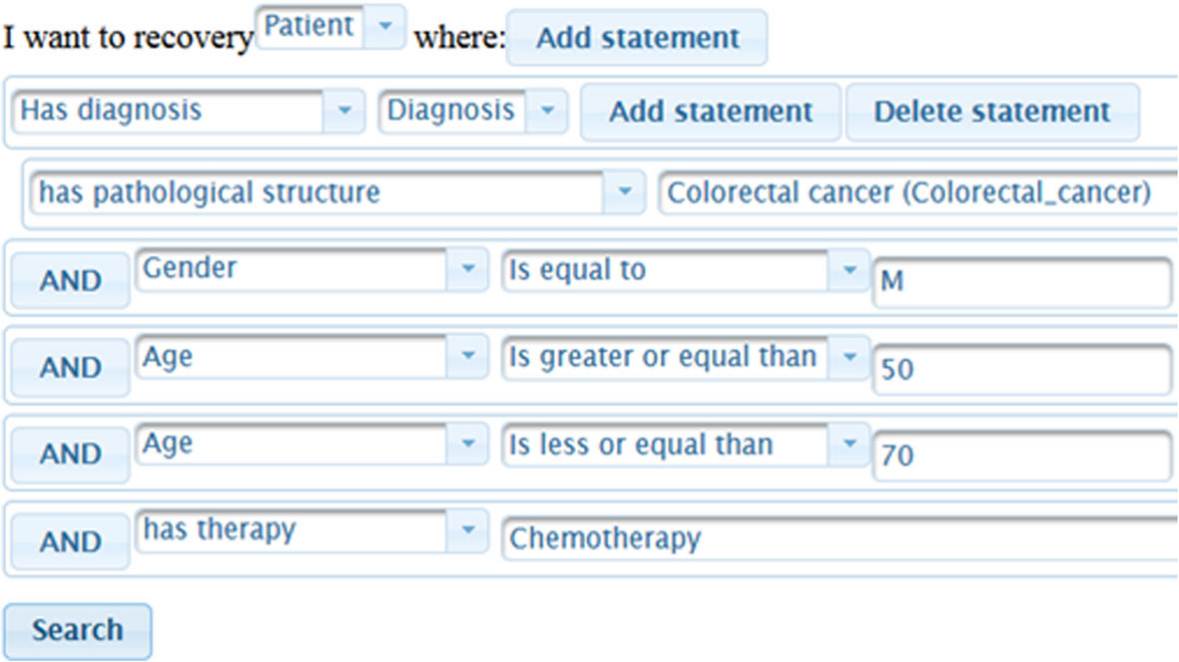
Ontology-driven searcher view

Figure 8 shows the selection and the aggregation of patients using the following criteria: male patients aged between 50 and 70, diagnosed with colorectal cancer, and who have received Chemotherapy. Table 4 shows the query generated for this case.

**Table 4:** SPARQL query generated by ODS for a filter

PREFIX ods: 〈http://www.imib.es/ontologies/disease-times 〉
SELECT DISTINCT ?s WHERE {
{?s rdf:type ?t FILTER (?t IN (ods:Patient))}.
{
{?s ods:hasDiagnosis ?a0.
{?a0 rdf:type ?ta0 FILTER (?ta0 IN (ods:Diagnosis))}
}. {?a0 ods:hasPathologicalStructure ?a01.
{?a01 rdf:type ?ta01 FILTER (?ta01 IN (ods:Colorectal_cancer))}
}. {?s ods:gender ?a1. FILTER (str(?a1) = ’M’)}.
{?s ods:age ?a2. FILTER (?a2 〉= 50)}.
{?s ods:age ?a3. FILTER (?a3 〈= 70)}.
{?s ods:hasTherapy ?a4.
{?a4 rdf:type ?ta4 FILTER (?ta4 IN (ods:Chemotherapy))}
}
}
}

**Fig. 9:**
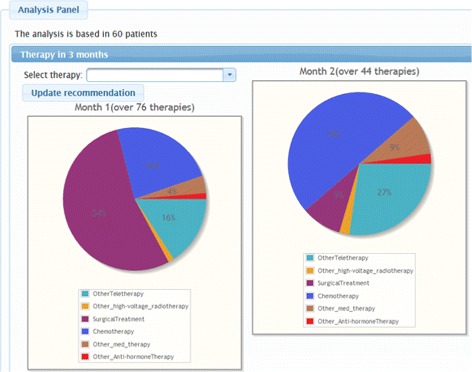
Excerpt of the aggregated disease timeline of a patient group

After the selection and the aggregation of patients, the system generates charts that contain the therapies and the disease courses of the patients. This service can be employed as an exploratory therapy simulator. Optionally, the entire time matrix can be recalculated by selecting a certain therapy. This can help the user to estimate which therapy is likely to be the most appropriate. Figure 9 shows an excerpt of the panel for analysing the first two months of the therapies of a group of 60 patients.

#### The enrichment analysis

Term enrichment was performed on several patient groups using the hypergeometric distribution method for the ICD10 code annotations on each diagnosis. First, we used a sample of cancer cases related to over 300 patients. Our design requirement for this sample was to include patients of both genders, so we discarded breast and prostate cancer for this analysis. The sample contained three main cohorts: diagnosis of lung cancer (469), diagnosis of melanoma (338) and diagnosis of colorectal cancer (311).

Table 5 shows the results associated with lung cancer for males and females. The results show that the difference between both groups is not significant for lung cancer but, as shown in Table 6, it is significant for colorectal cancer. For example, Malignant neoplasm of rectum is clearly over-represented in the gender male, which permits to conclude that this diagnosis is much more common in men.

**Table 5:** Term enrichment for ICD10 cores of Lung cancer

ICD 10 Code	*P*-value	*P*-value
	Male	Female
	(308)	(152)
C34.0 (Main bronchus)	0.77	0.35
C34.1 (Upper lobe, bronchus or lung)	0.42	0.77
C34.2 (Middle lobe, bronchus or lung)	0.48	0.58
C34.3 (Lower lobe, bronchus or lung)	0.71	0.45
C34.8 (Overlapping lesion of bronchus and lung)	0.51	0.63
C34.9 (Bronchus or lung, unspecified)	0.55	0.40

**Table 6:** Term enrichment for ICD10 cores of colorectal cancer

ICD 10 Code	*P*-value	*P*-value
	Male	Female
	(175)	(127)
C17.0 (Duodenum)	0.21	0.90
C17.1 (Jejunum)	0.31	0
C17.2 (Ileum)	0.94	0.47
C17.8 (Overlapping lesion of small intestine)	0	0.41
C17.9 (Small intestine, unspecified)	0.81	0.65
C18.0 (Caecum)	0.99	0.03
C18.1 (Appendix)	0.86	0.28
C18.2 (Ascending colon)	0.90	0.26
C18.3 (Hepatic flexure)	0.11	0.96
C18.4 (Transverse colon)	0.15	0.93
C18.5 (Splenic flexure)	0.59	0.79
C18.6 (Descending colon)	0.86	0.30
C18.7 (Sigmoid colon)	0.94	0.18
C18.9 (Colon, unspecified)	0.37	0.75
C19 (Malignant neoplasm of rectosigmoid junction)	0.96	0.18
C20 (Malignant neoplasm of rectum)	6.39E-4	0.99
C21.0 (Anus, unspecified)	0.98	0.18
C21.1 (Anal canal)	0.87	0.30
C21.8 (Overlapping lesion of rectum, anus and anal canal)	0	0.07
D01.0 (Colon)	0.81	0.65
D01.2 (Rectum)	0.56	0

#### Target users

The target users of our platform are described next:

- Physicians can use our platform to extract knowledge from the cancer registry in aggregated form filtering on the risk of patients by applying clinical criteria. Furthermore, they can obtain a graphical representation of the disease course of a concrete patient or a group of patients.
- Health managers can use our platform to generate customisable dashboards to prepare a follow-up of the clinical services involved in the diagnosis or therapies for cancer.
- Tumor documentaries can use the platform to detect cases with incomplete or inconsistent documentation for data curation.

## Discussion

Cancer registries have become a basic tool for disease research and treatment. Nowadays, there are several technological solutions able to manage and analyse the information of patients with a determined diagnosis. However, the lack of formal semantic models is a problem when personalised analyses or external data links are required. In this paper we have presented a semantic platform for the analysis and visualisation of records in an institutional cancer registry. Based on the analysis of requirements, we have developed an ontology that models the semantics of a regional cancer registry. We have used this model and SWIT for transforming and storing simulated data from a cancer registry in a semantic data store. Our approach permits users to formulate incremental, user-defined queries with a graphical user interface based on the ODS. The results of the queries can be displayed in several customisable ways, allowing for the generation of on-demand dashboards. The complex timelines of the disease of individuals and aggregated patients can be clearly represented.

Rule-based systems and logic-based models have been semantic approaches applied to cancer registries, such as analysis of cancer registry processes [7], quality assurance [8] and decision support [9]. Our approach innovates by combining traditional technologies such as relational databases and semantic web technologies. We have created an OWL ontology for representing some aspects of an institutional, local cancer registry. We have developed an RDF repository whose structure is driven by the OWL ontology and permits to work by exploiting the semantics of the content. In this way retrieval is semantically enabled, so that queries are independent of the relational data structures of conventional databases. Our technological infrastructure has permitted us to develop a semantic searcher for navigating through the complete cancer registry, to extract semantic profiles of the patients, and to analyse the structure of disease courses.

Our approach provides powerful and precise search capabilities assisted by a customisable dashboard adaptable to the requirements of each user. This proposal is very similar to the tools presented in [43], but we innovate by permitting users to generate re-usable templates. Furthermore, the templates do not only allow the generation of search forms but also of parameterised user-customisable dashboards. The platform permits to use the entities defined in the OWL ontology for creating the queries in a more intuitive way than using a traditional relational model. Furthermore, the use of a NoSQL database (e.g. RDF repository) allows to use a robust and scalable architecture for large clinical data warehouses [49]. Another important advantage of using semantic knowledge modelling is the possibility of sharing information and comparing clinical cases and processes.

The semantic profiles enable the generation of timelines for different patient records. Our approach combines multimedia and temporal visualisations [24] which can be customised by the users. The semantic profiles can be aggregated, hence enabling the generation of timelines of a patient group with similar characteristics. This visualisation can be used as a graphical representation of a Bayesian network. Clinicians can interact with the visualisation to discover likely courses of patients’ diseases. The platform offers data analysis based on term enrichment to support clinicians to generate groups of patients.

### Limitations

One limitation of this work is the application of a preliminary version of an ontology of cancer registry data. This ontology needs to be reviewed and extended. However, we believe that the OQuaRE quality scores of the ontology permit to use it for proof-of-concept implementations and experiments such as the one presented in this work.

Another limitation is the use of simulated data, because real data would enable a more reliable (1) validation of the correctness and completeness of the system, (2) testing of the performance of the system, and (3) evaluation of the impact of missing data in the performance [8].

In this work, we have been able to evaluate only some components of the platform. A complete evaluation would mean to measure the following metrics: efficiency, usability, usefulness of the graphical representations of the analysis of the disease courses or patients or group or patients and the capacity to develop new customizable dashboards by the users.

### Future work

The results of this work were shown in a clinical session of the epidemiological service of our largest regional hospital, the Virgen de la Arrixaca Hospital in Murcia, Spain. The physicians showed their interest in applying the same methodology to the Colorectal Cancer Prevention Program of the Region of Murcia (Spain). This use case will include real data from 322,869 patients recruited since 2006. Nowadays, the physicians can generate customisable dashboards^12^ and they are interested in a prediction of their future level of risk of patients.

In addition, a study combining real data from the Department of Epidemiology of Murcia Regional Health Council (Spain) and the cancer registry of the Comprehensive Cancer Center Freiburg (Germany) is planned. On the clinical side, this would permit to perform studies with data originating in different registries as well as to perform comparative studies on the characteristics and evolution of cancer patients in different populations or on clinical oncology practice in these regions. On the technical side, this would permit to exploit the fact that ontology-based approaches facilitate data integration. Although data integration has not been investigated in this work, we believe that sharing the same ontology for different registries would enable interoperability, and the data could be jointly exploited by means of distributed SPARQL queries. By the same means, they could also be used to create an integrated data warehouse. The decision between both implementation options depends on the requirements of the use case, is due to the time cost of executing the distributed queries and the effort needed to maintain the data warehouse. However, this effort does not imply major changes in the RDF data representation. Such a study could also test how the ontology copes with different registries, which we believe it is a relevant quality indicator for our ontology.

We plan to extend the platform with studies of other chronic pathologies, which might also include a clinical validation. In this way, we plan to apply the platform for monitoring clinical trials thanks to the flexibility of the ODS and the customisable dashboards. Furthermore, we plan to use *D3SPARQL* [60] to enrich the dashboard plots.

Finally, we would like to use this model to generate rules that serve to automatically generate patient groups or for quality assurance of the data.

## Conclusion

This work has demonstrated that ontologies and the RDF repositories can be effectively combined for exploiting a local cancer registry. On the one hand, we constructed an ontology that models the knowledge of local cancer registry. On the other hand, we have used semantic web technologies for building a platform to analyse the complex timelines of a patients with cancer. Besides, our semantic structure has allowed for representing the aggregated disease timelines of patient groups.

The semantic infrastructure has also permitted the generation of graphical representations of the stored knowledge in the cancer registry with the generation of customisable dashboards.

The work is an example of how ontologies can guide the entire life cycle of a analysis platform: data transformation, exploitation and knowledge generation. These technologies allow users to configure advanced searches, build custom dashboards and establish complex analysis from semantic profiles. Furthermore, semantic technologies establishes the bases to link to external data sources and comparative analysis with other organizations. We believe that this work provides new insights about how semantic technologies can be applied to the exploitation of clinical data in general, and to clinical registries in particular.

## Endnotes

^1^ http://www.elekta.com/healthcare-professionals/products/elekta-software/cancer-registry.html

^2^ http://www.oncolog.com/?cid=7

^3^ http://www.askcnet.org/

^4^ http://protege.stanford.edu/

^5^ http://www.ncri.ie/

^6^ http://virtuoso.openlinksw.com/dataspace/doc/dav/wiki/Main/http://virtuoso.openlinksw.com/dataspace/doc/dav/ http://virtuoso.openlinksw.com/dataspace/doc/dav/wiki/Main/wiki/Main/

^7^ http://commons.apache.org/proper/commons-math/

^8^ http://sele.inf.um.es/ontologies/cancer-registry2.owl

^9^ http://sele.inf.um.es/SECARE/

^10^ http://sele.inf.um.es/ontologies/individuals.zip

^11^ The test has been carried out in a local machine with MySQL 5 as relational database and Virtuoso 7 as RDF repository.

^12^ http://sele.inf.um.es/SECOLON/

## Abbreviations

ASA: American society of anesthesiologists
DL: Description logics
EMR: Electronic medical record
ICD: International classification of diseases
OBO: Open biomedical ontologies
ODS: Ontology-driven search
OQuaRE: Ontology quality evaluation framework
OWL: Web ontology language
PCS: Procedure coding system
RDF: Resource description framework
SNOMED CT: Systematic nomenclature of medicine - clinical terms
SPARQL: SPARQL protocol and RDF query language
SWIT: Semantic web integration tool
TNM: Classification of malignant tumours
RDFS: Resource description framework schema
